# Higher chronic stress and less satisfaction with GP communication in migrants with unemployment experience: data from the representative German DEGS1 and the GPCare-1 study

**DOI:** 10.1186/s12875-022-01691-1

**Published:** 2022-04-20

**Authors:** Luisa K. Offenberg, Samira T. Sommer, Manuela Schmidt, Stefanie Kasten, Florian Bockheim, Boris Gavrilov, Carmen Hunzelar, Nur Ikar, Maja P. S. Oberholz, Joana L. Paños-Willuhn, Birgitta Weltermann

**Affiliations:** Institute of General Practice and Family Medicine, University Hospital Bonn, University of Bonn, Venusberg-Campus 1, 53127 Bonn, Germany

**Keywords:** Physician-patient communication, Primary health care, Chronic stress, Migrant, Unemployment, General practitioner

## Abstract

**Background:**

The impact of unemployment on health is well studied. However, information on associations of unemployment, migration background and general practitioner-patient communication is scarce.

**Methods:**

Data from the representative German Health Interview and Examination Survey for Adults (DEGS1) of individuals in working age (*n* = 5938) were analysed stratified by unemployment and migration background. Using official weighting factors, the prevalence of chronic stress, having ≥1 chronic disease, having a GP and GP visits in the last 12 months was determined. Multivariate regression models were analysed for associations between unemployment, migration background, and other socio-demographic characteristics with GP visits and chronic stress. Data from the General Practice Care-1 (GPCare-1) study (*n* = 813 patients) were analysed for differences in patient-physician communication between unemployed with and without migration background. Reverse proportional odds models were estimated for associations of unemployment and migration background with physician-patient communication.

**Results:**

In the DEGS1, 21.5% had experienced unemployment (*n* = 1170). Of these, 31.6% had a migration background (*n* = 248). Compared to unemployed natives, unemployed with migration background had higher chronic stress (mean: 14.32 vs. 13.13, *p* = 0.02), while the prevalence of chronic disease was lower (21.7% vs. 30.2%, *p* = 0.03). They were less likely to have a GP (83.6% vs. 90%, *p* = 0.02), while GP visits were similar (mean: 3.7 vs. 3.3, *p* = 0.26). Migration background and unemployment experience were not associated with GP visits, while both factors were significantly associated with higher chronic stress (both: *p* < 0.01). In GPCare-1, 28.8% had ever experienced unemployment (*n* = 215). Of these, 60 had a migration background (28.6%). The unemployed with migration background reported less frequently that the GP gives them enough space to describe personal strains (46.5% vs. 58.2%; *p* = 0.03), and that their problems are taken very seriously by their GP (50.8% vs. 73.8%; *p* = 0.04). In multivariate analyses, migration background showed a lower probability of having enough space to describe personal strains and feeling that problems were taken very seriously.

**Conclusion:**

Unemployment experience and migration background were associated with higher chronic stress. Only migration background was associated with less satisfaction regarding physician-patient communication.

## Key-points

- Experience of unemployment in individuals with migration background is a growing public health concern in Germany as migrant populations are increasing.
- Individuals with migration background and unemployment experience were identified as risk group for high chronic stress.
- Individuals with migration background and unemployment experience were more likely to report that their GP did not take their problems seriously and were not given enough space to address personal strains.
- To improve physician-patient communication on social problems of individuals with migration background, culturally sensitive trainings as well as strategies to inform and encourage migrant populations are needed.
- Health care policy should conceptualize, develop, finance and implement services that allow for profound understanding and support of migrant populations by GPs and community services.

## Introduction

In 2013, the International Labour Organization defined persons in unemployment as “all those of working age who were not in employment, carried out activities to seek employment during a specified recent period, and were currently available to take up employment given a job opportunity” [1]. In July 2020, 7.4% unemployed individuals were registered in Europe, and 4.4% in Germany respectively [2]. The Organisation for Economic Cooperation and Development (OECD) recently showed for Europe that foreign-born individuals were more frequently unemployed than natives [3]. These findings are a public health concern since unemployment was shown to be associated with a higher prevalence of chronic illnesses and mortality [4, 5]. Especially, higher rates of adverse mental outcomes such as depressive disorders, anxiety, insomnia and stress were shown [6, 7]. Based on 41 studies, a review by Norström et al. (2014) showed that most studies found a negative effect of unemployment on health but additional studies differentiating between certain subgroups are needed, e.g. on individuals with migration background [8].

Scarce data on the impact of unemployment on the health of individuals with migration background showed for example that migrant men experienced the highest decrease compared to both males and females with employment in life satisfaction when becoming unemployed [9]. Furthermore, health status in unemployed migrants varies by country of origin, e.g., unemployed Turkish women, especially at high age, experiencing high levels of distress [10]. Given the current increase in migration populations, a better understanding of health outcomes in various migrant populations and the role of the health care system, especially primary care, is needed. In Germany alone the number of migrants increased between 2011 and 2016 from 6.3 to 9.2 million increasing the prevalence from 7.9 to 11.2% within the general population [11].

General practitioners are relevant contact persons for individuals of all ages and backgrounds. Therefore, they have a strong position to address not only stress and chronic illnesses but also social issues such as unemployment. Although Zimmermann et al. (2018) showed that 43.3% of GPs were consulted at least three times a week by patients with work/unemployment problems [12], data is limited regarding subpopulations with migration background. So far, research described differences between natives and individuals of different ethnicities in physician-patient communication in general [13, 14], but did not specifically address physician-patient communication regarding personal strains.

This study builds on data from the nationally representative German Health Interview and Examination Survey for Adults (DEGS1) and the General Practice Care-1 patient survey (GPCare-1). Using descriptive and multivariate analyses, it investigates associations between various socio-demographic parameters including unemployment experience and migration background with the number of GP visits in 12 months and the patient-reported quality of GP-patient communication.

## Methods

### Study design

The study draws on data from two surveys:

1. the representative German Health Interview and Examination Survey for Adults (DEGS1),
2. the General Practice Care 1 study (GPCare-1).

#### German health interview and examination survey for adults (DEGS1)

The DEGS1 study which is representative for the general German population was conducted by the Robert Koch Institute as part of the German health monitoring system (2008–2011). The DEGS1 study has a mixed design which allows for cross-sectional and longitudinal analyses. The study population was sampled by inviting participants according to the study protocol additionally to participants who had already participated in the German National Health Interview and Examination Survey 1998 (GNHIES98). The DEGS1 study included a total of 8152 participants. 3959 participants had already been part of the GNHIES98 while 4193 participants were newly recruited. In this paper participants were only included if data on experience of unemployment during the last 5 years was available; ergo, the study population in this paper consists of 5938 participants of working age (18 to 64 years old). More details regarding the study protocol of the DEGS1 can be found elsewhere [15]. The DEGS1 data used for this analysis were kindly provided by the Robert Koch Institute as public use file.

The following DEGS1 measurements of socio-demographic parameters were used for the analyses:

- Age (in years)
- Sex (male, female)
- Education level classified according to the International Standard Classification of *Education* [16]*.*
- Socioeconomic status (SES) was calculated based on information regarding education, employment status and income, which was subsequently classified into low, middle and high socioeconomic status (for details see [17]).
- Number of underage individuals in household
- Social support determined by the Oslo-3-Items-Social-Support Scale (Oslo-3) classified in three categories: low (3–8 points), middle (9–11 points), high (12–14 points) [18, 19].
- Financial dependency was determined by asking participants if they were the main breadwinner of the household.

Issues of migration and employment were addressed using the following items:

- Migration background was defined as the participant or at least one parent was born outside of Germany.
- Migration generation (first or second)
- Nationality
- Self-assessed knowledge of the German language
- Current employment status (employed, unemployed)
- Unemployment in the past 5 years (yes, no)
- Length of unemployment during the past 5 years

In addition, measurements of chronic stress, illness and GP contacts were used for the analyses:

- Chronic stress was determined by the 12-item Screening Scale of the Trier Inventory for the Assessment of Chronic Stress (TICS-SSCS) which encompasses chronic worrying, work related und social overload, excessive demands and lack of social recognition in the past 3 months [20, 21]; the sum score was classified into below average to average (0–11 points), above average (12–22 points) and high stress (23–48 points) [22].
- Having at least one chronic illness (e.g., diabetes, heart disease)
- Having a GP (yes, no)
- Self-reported number of GP visits in the last 12 months

#### GPCare-1 study addressing patients’ communication with their GP

Data collection for the General Practice Care 1 study (GPCare-1) was conducted from June until August 2020 in 12 primary care offices in the Greater Bonn region, Germany. The participating primary care offices belong to the academic teaching practice network associated with the Institute of General Practice and Family Medicine, University Hospital Bonn. In each participating practice, all adult patients who visited the practice during the time of recruitment were asked to participate. Patients were eligible if they had sufficient language skills and were mentally capable to fill the self-administered questionnaire in German, English, Turkish or Arabic. 813 participants between 18 and 91 years old filled out the questionnaire.

### GPCare-1: questionnaire design

To allow for comparison, the GPCare-1 questionnaire used the DEGS1-questions on age, migrant status and gender, except that the third gender (diverse) was added. Participants were asked for their highest level of education. Education level was then computed into three categories: low education (no school education/ secondary school up to 9th/up to 10th grade), middle education (high school (A-levels)/vocational school) and high education (university degree).

As no validated screening tool for patient-physician communication on social problems is available in German, eight questions were constructed based on existing questionnaires: the Patient Reactions Assessments Instruments (PRAD) [23], the Medical Interview Satisfaction Scale (MISS) [24], the patient requests form [25] and the patient-doctor relationship questionnaire (PDRQ-9) [26]. The first four questions addressed patients’ experiences with their GP, the second four questions focussed on patients’ preferences regarding their GP contact. Details on questionnaire items are provided in Table 5. All items used a five option Likert-type answer (strongly disagree to strongly agree). The questionnaire was piloted by 40 individuals of the German general population with minor revisions thereafter.

### Statistical analysis

In the DEGS1 population, all analyses were carried out using weighted complex samples procedures to allow for conclusions representative of the German general population. The standardized weighting factor was provided by the Robert Koch Institute and took age, gender, nationality, education, population for each federal state and BIK classification details into account. To allow weighting of participants who had already participated in 1998 the re-participation probability was estimated using logistic regression. For further details please see [27]. The number of cases is reported unweighted while the prevalence and the confidence interval is weighted. In Table 1 the experience of unemployment in the past 5 years was used as filter variable to stratify subpopulations. Weighted chi-squared tests and t-tests were applied. In Table 2 DEGS1 participants were filtered for unemployment experience in the past 5 years (*n* = 1170) and stratified by migration background. Weighted chi-squared tests for categorical data as well as t-tests for mean values were applied to determine differences between individuals with and without migration background. Considering all participants, one multiple linear regression was performed using migration background, unemployment experience, chronic stress and chronic illness as independent variables and GP visits as dependent variable. Age, gender and socioeconomic status were used as covariates. Outliers who had reported > 14 GP visits (*n* = 100; 1.7%) were excluded to meet the assumptions of the multiple linear regression. In 252 participants no data on the number GP visits was available so these cases were also excluded from the analysis (Table 3). Additionally, a poisson analysis was performed using the same independent variables as in the multiple linear regression and chronic stress (TICS score) as dependent variable (Table 4). To account for missing values multiple imputation with chained equations with 25 iterations and repetitions was carried out [28].

In the GPCare-1 dataset, descriptive analyses were performed. In the subpopulation of individuals with unemployment experience during their lifetime (*n* = 215), information on migration background was available in 210 cases. Chi-square tests and t-tests were used to determine differences between individuals with and without migration background (Table 5). For the multivariate models, two cases with diverse gender were excluded as the number of cases did not allow for subgroup analyses. The first multivariate model analysed for associations of the dependent variable “My doctor gives me enough space to describe personal strains” with gender, age, migration background, chronic disease, unemployment status, stress and education level (Table 6). The second model estimated the relationship between the dependent variable “I get the feeling that my doctor takes my problems very seriously” and the same covariates (Table 7). Both models were estimated as a reverse proportional odds model that takes the ordinality of the response scale into account: the probability of observing at least one category (e. g. partial or full agreement) was estimated [29]. Missing values were taken into account by applying multiple imputation with chained equations with 25 iterations and repetitions [28].

In all analyses, statistical significance was set at *p* < 0.05 (two-tailed). Analyses were conducted using IBM Statistical Package for Social Sciences (SPSS 25.0) for Windows (IBM Corp., Armonk, NY, USA). Poisson and proportional odds regression models were calculated with R (Version 4.1.2).

### Ethics

The Charité-Universitätsmedizin Berlin Ethics’ Committee had provided ethical approval for the DEGS1 study protocol in September 2008 (No. EA2/047/08) [15]. The Ethics’ Committee of the Medical Faculty of the University of Bonn approved the study protocol for GPCare-1 in June 2020 (Ref. No. 215/20). All participating patients received verbal and written information on study procedures, anonymity, and confidentiality. Also, they were informed that participation was voluntary. No formal written consent was required as the return of the anonymous questionnaire indicated informed consent from the patient for their data to be used in the study. The GPCare-1 study was registered in the German Clinical Trials Register (DRKS00022330). Both studies were conducted in accordance with the 1964 Helsinki declaration and its later amendments or comparable ethical standards.

## Results

### Characteristics of the DEGS1 population of working age (Table 1)

**Table 1:** DEGS1 population in working age: Characteristics of participants stratified by unemployment experience (past 5 years)

	Total sample (*N* = 5938)			Individuals with unemployment experience in the past five years (*N* = 1170)			Individuals without unemployment experience in the past five years (*N* = 4490)			*p*-value
	N^a^	%^a^	95% CI	N^a^	%^a^	95% CI	N^a^	%^a^	95% CI	
**Sociodemographic**										
Age mean (standard deviation)	41.63 (13.06)		41.29–41.97	39.53 (12.66)		38.65–40.41	41.94 (13.13)		41.52–42.36	< 0.01
Gender (female)	3149	49.4	47.8–51.1	573	45.2	41.4–49.1	2417	50.1	48.3–51.9	0.02
Socioeconomic status										< 0.01
- Low	847	18.0	16.6–19.6	319	30.6	27.2–34.1	468	13.4	12.0–14.9	
- Middle	3521	60.6	58.9–62.3	674	56.5	52.7–60.3	2730	62.4	60.5–64.2	
- High	1506	21.4	19.7–23.1	177	12.9	10.5–15.7	1289	24.2	22.4–26.3	
Number of underage individuals living in household, mean (standard deviation)	0.57 (0.90)		0.54–0.61	0.54 (0.9)		0.47–0.62	0.58 (0.9)		0.55–0.62	n.s.
Main breadwinner	3061	53.1	51.5–54.7							
**Migration**										
Migration background	900	21.6	19.4–24.0	248	31.6	27.6–36.0	622	18.5	16.4–20.9	< 0.01
First generation migrant	553	67.3	63.3–71.0							
Non-German nationality	390	12.2	10.4–14.3							
Self-reported little/poor knowledge of German	42	9.5	7.0–12.8							
**Unemployment**										
Currently unemployed	372	6.9	5.9–8.0							
Was unemployed in past 5 years	1170	21.5	19.9–23.2							
**Health outcomes**										
Chronic stress, mean (standard deviation)	12.11 (7.98)		11.85–12.38	13.51 (8.72)		12.86–14.15	11.73 (7.70)		11.45–12.01	< 0.01
Chronic stress categorical										< 0.01
Below to average				548	44.9	41.2–48.6	2347	52.8	50.9–54.6	
Above average				453	38.9	34.9–43.1	1683	37.8	36.1–39.5	
High				158	16.2	13.6–19.3	445	9.4	8.5–10.5	
At least one chronic disease	1501	24.4	23.0–26.0	329	27.8	24.6–31.3	1105	23.5	21.8–25.2	0.02
Has a general practitioner	5261	89.1	87.6–90.4	1049	87.7	84.6–90.3	4042	89.5	88.1–90.8	n.s.
- Number of GP visits during last 12 months, mean (standard deviation)	2.99 (4.54)		2.80–3.19	3.41 (5.06)		2.93–3.88	2.84 (4.13)		2.67–3.01	< 0.01

The sociodemographic and health characteristics of DEGS1 participants of working age (18–64 years old) were compared of individuals with and without experience of unemployment in the past 5 years

^a^n or percent unless noted otherwise

The DEGS1 data set included 5938 individuals of working age (18–64 years old). Their mean age was 42 years. Female and male participants were equally represented, and more than half of the participants had a medium socio-economic status (60.6%).

Of the participants, a total of 1170 (21.5%, CI: 19.9–23.2) had a history of unemployment during the past 5 years, while 372 (6.9%, CI: 5.9–8.0) were currently unemployed. A total of 900 participants (21.6%, CI: 19.4–24) had a migration background, the majority (*n* = 553, 67.3%, CI: 63.3–71) being first generation migrants. The most frequent foreign nationalities were Turkish (19.6%, CI: 14.6–25.7), Italian (9.6%, CI: 6.3–14.3), Polish (6%, CI: 3.8–9.3), Croatian (5.1%, CI: 2.9–8.8) or Austrian (4.4%, CI: 2.4–7.8).

#### Comparison of subpopulations stratified by unemployment experience (Table 1)

When comparing individuals with and without unemployment experience, the results showed that participants with unemployment experience had a higher prevalence of being younger (mean 39.5 years vs. mean 42 years, *p* < 0.01), male (female: 45.2% vs. 50.1%, *p* = 0.02), low SES (30.6% vs. 13.4%, *p* < 0.01), and migration background (31.6% vs. 18.5%, *p* < 0.01). With regard to health outcomes, participants with unemployment experience had a higher level of chronic stress (13.51 vs. 11.73, *p* < 0.01), and a higher prevalence of having at least one chronic disease (27.8% vs. 23.5%, *p* = 0.02). While the subgroups did not differ regarding having a GP, participants with unemployment experience visited their GP more often during the last 12 months (3.41 vs. 2.84, *p* < 0.01).

#### Comparison of subpopulations with unemployment experience stratified by migration background (Table 2)

**Fig. 1:**
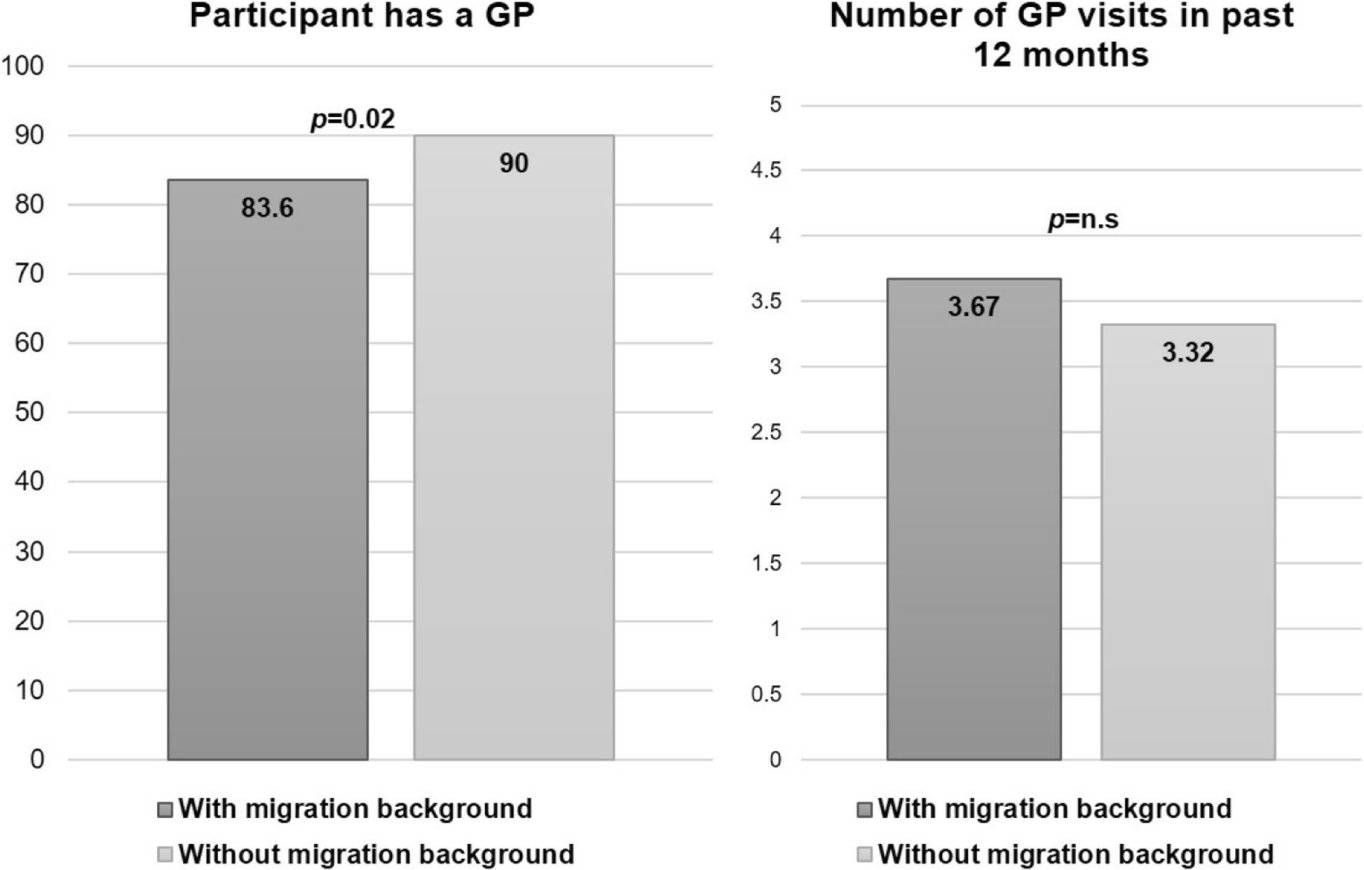
DEGS1: Prevalence of having a GP and number of GP contacts in participants with unemployed experience in last 5 years, stratified by migration background

Information on migration background was available for 98.7% of those with a history of unemployment (1155 of 1170; missing 15 (1.3%)). Of these, 31.6% (*n* = 248) had a migration background. Compared to natives with unemployment experience, participants with migration background and a history of unemployment were significantly younger (36.9 vs. 40.7 years, *p* < 0.01), were more likely to have under-aged individuals in their household (0.75 vs. 0.44, *p* < 0.01), and to have a low educational level (31.9% vs. 13.6%, *p* < 0.01). The two subgroups did not differ regarding current unemployment rates (27.1% vs. 28.6%, n.s.), the average months of unemployment in the past 5 years (15.76 vs. 16.51, n.s.) and social support (10.23 vs. 10.47, n.s.). Regarding health outcomes, the subpopulation with migration background showed a significantly higher chronic stress level (14.32 vs. 13.13, *p* = 0.02) while the prevalence of having at least one chronic disease was lower (21.7% vs. 30.2%, *p* = 0.03). The migrant subpopulation was significantly less likely to have a GP (83.6% vs. 90%, *p* = 0.02), while the mean number of GP visits in the last year did not differ between groups (3.67 vs. 3.32, *p* = 0.26). For details see Fig. 1.

**Table 2:** Sociodemographic and health characteristics of unemployed DEGS1 participants, stratified by migration background (*n* = 1170)

Missing = 15	Individuals with migration background (*N* = 248)			Natives (*n* = 907)			*p*-value
	N^a^	%^a^	95% CI	N^a^	%^a^	95% CI	
Individuals who have experienced unemployment in the past five years	248	31.6	27.6–36.0	907	68.4	64.0–72.4	< 0.01
**Sociodemographic**							
Age, mean (SD)	36.89 (12.56)		35.20–38.58	40.72 (12.56)		39.73–41.71	< 0.01
Gender (female)	120	43.3	35.1–51.9	444	45.8	41.9–49.8	n.s.
Socioeconomic status							n.s.
- Low	82	34.7	28.0–42.2	229	27.6	23.9–31.8	
- Medium	133	55.3	47.8–62.6	534	57.8	53.4–62.0	
- High	33	9.9	6.1–15.8	144	14.6	11.6–18.2	
Number of underage individuals, mean (SD)	0.75 (1.071)		0.58–0.92	0.44 (0.797)		0.37–0.52	< 0.01
Education							< 0.01
- Low	66	31.9	25.6–39.0	90	13.6	10.7–17.2	
- Medium	114	47.8	40.4–55.4	604	67.4	62.9–71.5	
- High	68	20.2	15.0–26.8	213	19.0	15.8–22.7	
**Unemployment**							
Currently unemployed	64	27.1	20.5–34.8	248	28.6	24.8–32.6	n.s.
Months unemployed in the past 5 years mean (standard deviation)	15.76 (17.88)		12.95–18.57	16.51 (17.36)		14.93–18.08	n.s.
**Social conditions**							
Main breadwinner	111	48.0	40.3–55.7	475	53.3	49.0–57.5	n.s.
Social support mean (standard deviation)	10.23 (2.11)		9.89–10.57	10.47 (2.06)		10.30–10.64	n.s.
Social support categorical							n.s.
- Low	43	18.5	13.1–25.5	137	16.4	13.4–19.9	
- Medium	133	53.9	45.9–61.8	456	50.5	46.6–54.3	
- High	71	27.5	21.2–35.0	308	33.1	29.6–36.8	
**Health outcomes**							
Chronic stress mean (standard deviation)	14.32 (9.36)		12.73–15.91	13.13 (8.38)		12.45–13.81	0.02
Chronic stress categorical							n.s.
- Below to average	109	42.4	34.9–50.3	432	45.9	41.8–50.1	
- Above average	96	37.2	29.7–45.4	353	40.0	35.7–44.4	
- High	39	20.4	14.2–28.3	115	14.1	11.5–17.1	
At least one chronic disease	66	21.7	16.5–28.1	258	30.2	26.2–34.5	0.03
Has a GP	216	83.6	77.1–88.5	822	90.0	86.7–92.6	0.02
Number of GP visits in last year, *mean, SD*	3.67 (6.35)		2.56–4.78	3.32 (4.38)		2.87–3.77	n.s.

^a^n or percent unless noted otherwise

#### Associations of migration background and unemployment experience with GP visits and chronic stress (Tables 3 and 4)

The multiple linear regression analysis showed no significant association of migration background and unemployment experience with the number of GP visits when controlling for covariates. Higher chronic stress, female gender, lower SES, and having a chronic illness were significantly associated with a higher number of GP visits. For details, please see Table 3.

**Table 3:** DEGS1 participants: Multivariate regression model on associations of sociodemographic and medical characteristics with GP visits (*n* = 5586)

Parameter	Estimate	Std. Error	95%-CI	*p*-value
Constant	2.317	0.201	1.921–2.714	< 0.01
Migration background (Ref. no migration background)	−0.170	0.111	− 0.390-0.049	0.127
Unemployment experience in the past five years (Ref. no unemployment experience in the past five years)	0.109	0.114	−0.116-0.334	0.339
Age (in years)	0.002	0.004	−0.005-0.009	0.498
Female (Ref. Male)	0.312	0.080	0.154–0.470	< 0.01
SES Score	−0.074	0.011	−0.096--0.053	< 0.01
Chronic stress (TICS score)	0.032	0.006	0.021–0.044	< 0.01
Has chronic illness (Ref. no chronic illness)	1.903	0.121	1.665–2.141	< 0.01
R^2^ Adj.	13.4%			

Being female, younger, having a chronic illness, migration background, unemployment experience in the past 5 years and having a lower SES were all significantly associated with chronic stress (*p* < 0.01). For details, please see Table 4.

**Table 4:** DEGS1: Multivariate model (Poisson analysis) on sociodemographic and medical characteristics associated with chronic stress (*n* = 5938)

Parameter	Rate	95%-CI	*p*-value
Female (ref. Male)	1.186	1.167–1.205	< 0.01
Age (in years)	0.999	0.998–0.999	< 0.01
Chronic illness (Ref. has no chronic illness)	1.127	1.098–1.156	< 0.01
Has a migration background (Ref. no migration background)	1.063	1.036–1.091	< 0.01
Unemployment experience in the past five years (Ref. no unemployment experience in the past five years)	1.099	1.074–1.124	< 0.01
SES Score	0.981	0.978–0.984	< 0.01

### GPCare-1: characteristics of the study population

A total of 813 patients participated in the data collection: their mean age was 51.6 years (SD ± 18.7 years), 59.3% were females, and the majority of participants had medium education (low 32.0%, medium 43.5%, high 24.6%). Data on migration background and unemployment experience was available for 724 participants (89.1%) Of these, 210 (29.0%) had experienced unemployment during their lifetime, the mean age was 47.92 years, 58.9% were female (*n* = 122), and 28.6% (*n* = 60) had a migration background. Compared to natives, patients with migration background and unemployment experience were younger (41.5 vs. 50.5 years, *p* < 0.01) and had a higher educational level (33.9% vs. 18.2%, *p* = 0.05).

#### GPCare-1: communication experience and preferences of patients with unemployment experience

Compared to natives, patients with migration background and unemployment experience had a significantly higher prevalence of strongly disagreeing with the statement that their GP takes their problems very seriously (migration background: 8.8% vs. natives: 3.5%, *p* = 0.04) and that the doctor gives them enough space to describe personal strains (migration background: 17.2% vs. natives: 4.3%; *p* = 0.03). Regarding the other six communication items, no significant differences between subgroups with and without migration background were found.

**Table 5:** GPCare-1**:** Sociodemographic characteristics and GP communication experiences stratified by migration background (*n* = 813)

	Patient population (*n* = 813)		Patients with migration background and unemployment experience (*n* = 60)		Natives with unemployment experience (*n* = 150)		*p*-value
	N	%	N	%	N	%	
**Sociodemographic**							
Gender							n.s.
- Female	474	59.3	32	53.3	90	61.2	
- Divers	2	0.3	0	0	1	0.7	
Age mean (standard deviation) ^a^	51.61 (18.67)		41.53 (12.56)		50.50 (14.58)		< 0.01
Education							0.05
- Low	247	32	19	33.9	56	37.8	
- Medium	336	43.5	18	32.1	65	43.9	
- High	190	24.6	19	33.9	27	18.2	
**Unemployment**							
Currently unemployed	42	5.8	10	18.5	20	14.8	n.s.
Experience of unemployment (including currently unemployed)	215	28.8					
**Migration**							
Migration background	194	25					
**Communication experiences**							
My doctor asks me about stress caused by personal strains							n.s.
- Strongly agree	212	28.8	19	32.8	32	23.2	
- Agree	180	24.5	12	20.7	35	25.4	
- Neutral	167	22.7	11	19.0	34	24.6	
- Disagree	122	16.6	9	15.5	28	20.3	
- Strongly disagree	54	7.3	7	12.1	9	6.5	
My doctor gives me enough space to describe personal strains ^a^							0.03
- Strongly agree	274	37.4	14	24.1	50	35.5	
- Agree	182	24.9	13	22.4	32	22.7	
- Neutral	160	21.9	15	25.9	42	29.8	
- Disagree	78	10.7	6	10.3	11	7.8	
- Strongly disagree	38	5.2	10	17.2	6	4.3	
My doctor makes me feel comfortable talking about sensitive things							n.s.
- Strongly agree	286	39.7	16	29.1	53	37.6	
- Agree	192	26.7	13	23.6	38	27.0	
- Neutral	142	19.7	14	25.5	31	22.0	
- Disagree	66	9.2	7	12.7	10	7.1	
- Strongly disagree	34	4.7	5	9.1	9	6.4	
I get the feeling that my doctor takes my problems very seriously ^a^							0.04
- Strongly agree	343	46.4	19	33.3	66	46.8	
- Agree	186	25.2	10	17.5	38	27.0	
- Neutral	122	16.5	15	26.3	21	14.9	
- Disagree	62	8.4	8	14.0	11	7.8	
- Strongly disagree	26	3.5	5	8.8	5	3.5	
**Communication preferences**							
I rather overcome personal strain without help from my doctor							n.s.
- Strongly agree	160	22.0	14	25.0	32	22.7	
- Agree	221	30.4	14	25.0	42	29.8	
- Neutral	211	29.1	18	32.1	35	24.8	
- Disagree	92	12.7	8	14.3	25	17.7	
- Strongly disagree	42	5.8	2	3.6	7	5.0	
Discussing personal strain with my doctor makes me uncomfortable							n.s.
- Strongly agree	84	11.6	5	8.8	19	13.6	
- Agree	163	22.5	19	33.3	27	19.3	
- Neutral	166	22.9	13	22.8	31	22.1	
- Disagree	193	26.7	15	26.3	40	28.6	
- Strongly disagree	118	16.3	5	8.8	23	16.4	
I would prefer my doctor to ask me directly about personal strains							n.s.
- Strongly agree	159	21.9	13	22.8	34	24.5	
- Agree	151	20.8	15	26.3	27	19.4	
- Neutral	197	27.1	16	28.1	43	30.9	
- Disagree	139	19.1	10	17.5	23	16.5	
- Strongly disagree	80	11.0	3	5.3	12	8.6	
I would prefer my doctor to give me a questionnaire regarding personal strains							n.s.
- Strongly agree	98	13.5	10	17.2	12	8.5	
- Agree	112	15.5	13	22.4	27	19.1	
- Neutral	117	16.2	11	19.0	23	16.3	
- Disagree	188	26.0	16	27.6	38	27.0	
- Strongly disagree	209	28.9	8	13.8	41	29.1	

The sociodemographic characteristics and patients’ communication experiences with GPs *n* = 813) were stratified by natives and individuals with migration background

^a^denotes a *p*-value < 0.05 between results of original variables of natives and individuals with migration background with unemployment experience

^b^n or percent unless noted otherwise

As outlined in Table 6 the multivariate results estimated that the probability of agreeing to the statement “My doctor gives me enough space to describe personal strains” was lower in individuals with migration background. However, having unemployment experience or not did not significantly change the probability of agreeing to the statement mentioned above.

**Table 6:** GPCare-1 (multivariate analysis): Relation of patient characteristics with “enough space to describe personal strains” (*n* = 811)

Parameter	Odds Ratio	95%-CI	*p*-value
Male (Ref. female)	0.98	0.75–1.28	n.s.
Age (in years)	1.01	1.00–1.02	n.s.
Has chronic illness (Ref. no chronic illness)	1.07	0.78–1.47	n.s.
Migration background (Ref. no migration background)	0.69	0.51–0.95	0.02
Unemployment experience (Ref. no unemployment experience)	0.76	0.55–1.05	n.s.
Chronic stress	0.99	0.97–1.00	n.s.
Secondary modern school (Ref. no schooling completed)	0.78	0.31–1.99	n.s.
O-levels (Ref. no schooling completed)	0.67	0.25–1.77	n.s.
High school degree (Ref. no schooling completed)	0.89	0.34–2.29	n.s.
Vocational degree (Ref. no schooling completed)	0.73	0.29–1.80	n.s.
University degree (Ref. no schooling completed)	0.59	0.23–1.47	n.s.

Regarding the statement “I get the feeling that my doctor takes my problems very seriously” both migration background and having higher stress decreased the chances of agreeing to this statement. Having unemployment experience or not did not show significant results. For more details, please see Table 7.

**Table 7:** GPCare-1 (multivariate analysis): Relation of patient characteristics with “doctor takes my problems very seriously” (*n* = 811)

Parameter	Odds Ratio	95%-CI	*p*-value
Male (Ref. female)	0.90	0.68–1.19	n.s.
Age (in years)	1.01	1.00–1.02	n.s.
Has chronic illness (Ref. no chronic illness)	1.16	0.84–1.61	n.s.
Migration background (Ref. no migration background)	0.70	0.51–0.97	0.03
Unemployment experience (Ref. no unemployment experience)	0.94	0.68–1.30	n.s.
Chronic stress	0.98	0.96–0.99	< 0.01
Secondary modern school (Ref. no schooling completed)	0.53	0.20–1.42	n.s.
O-levels (Ref. no schooling completed)	0.62	0.23–1.71	n.s.
High school degree (Ref. no schooling completed)	1.00	0.37–2.76	n.s.
Vocational degree (Ref. no schooling completed)	0.58	0.22–1.50	n.s.
University degree (Ref. no schooling completed)	0.57	0.22–1.49	n.s.

## Discussion

Based on the nationally representative DEGS1 data, our study showed a higher prevalence of chronic stress in individuals with migration background compared to natives with unemployment experience in the past 5 years. Higher chronic stress was significantly associated with unemployment experience and migration background. These findings are in line with results of Aichberger (2012), who observed higher distress levels in Turkish female migrants with unemployment experience in comparison to unemployed native women living in Berlin, Germany [30]. In contrast, researchers from Sweden reported no difference in the level of psychological distress between unemployed natives and migrants [31]. The contradicting results might be due to differences of the populations studied. In the current study population, most individuals with migration background and without a German nationality had a Turkish nationality. Study participants of the Swedish study included asylum seekers as well as immigrants from countries with guest worker’ programs [31]. Differences could also be due to diverging employment policies in Sweden and Germany [32].

Examining chronic stress in individuals with migration background is especially important since evidence from general practice offices in Israel has shown that general practitioners often missed to identify psychological distress in migrants [33]. These shortcomings are likely due to reasons on behalf of patients as well as physicians and the health care systems. For example, Schouten et al. (2006) showed that patients belonging to an ethnic minority tended to be less assertive and doctors seem to be interacting less with patients of ethnic minorities [14]. In the GPCare-1 study, patients with a migration background were less likely to report enough space to talk about psychosocial problems with their GP and that their problems were taken seriously. To improve communication quality for migrant patients various approaches were shown to be effective, but are not necessarily available widespread, e.g., trainings for general practitioners in multicultural communication and social problems, resources for medical interpreters and social workers [34–36]. Also, migrants should be informed and encouraged to speak up in GP communication whom they might view as authority rather than personal resource [37]. This study did not show any difference in GP visits for unemployed individuals with and without migration background. This finding adds to the diverging picture of GP utilization by migrants as reported by Graetz et al. [38]. A study conducted by Glaesmer et al., which showed a higher number of GP visits by first generation migrants compared to natives in Germany, did not focus on the subgroup of individuals with unemployment experience which might explain the differences [39].

### Strengths and limitations

The main topic of this study is novel in Germany, especially regarding chronic stress and physician-patient communication of migrant populations. Due to the cross-sectional design no causal relationships can be determined. Although nationally representative overall due to survey-specific weighting factors, it is recognized that the share of young men with migration background within the DEGS1 was higher than in the micro census [40]. For both subpopulations (unemployed with and without migration background) the DEGS1 question on unemployment in the last 5 years did not differentiate between job seekers directly after training and later in life. Data collection for the GPCare-1 study was conducted during the Covid-19 pandemic which might have led to higher chronic stress in patients. GPCare-1 data on GP-patient communication is used to complement the DEGS1 findings but does not allow for direct comparison as only the DEGS1 data are representative for the German population.

## Conclusions and perspectives

This study aims at increasing awareness on the association of unemployment and chronic stress in individuals with migration background. Migrants’ perception of GP contacts are reassuring for the German health care system, but point towards a potential for improvement. Future research, also using qualitative approaches, will help to better understand the complex interactions between migrants and GPs.

## Data Availability

The DEGS1 data set underlying this article were provided by the ‘Health Monitoring’ Research Data Centre at the Robert Koch Institute (RKI), which is accredited by the German Data Forum according to uniform and transparent standards (http://www.ratswd.de/en/data-infrastructure/rdc). Data are accessible on application to interested scientists for anonymous scientific secondary analyses. Detailed information on access, application forms, and guidelines can be obtained from datennutzung@rki.de. The data set of the GPcare-1 patient study will be shared on reasonable request to the Institute of General Practice and Family Medicine of the University of Bonn, Germany.
